# Supplementary material for: Characterization of Histone Modifications in Late-Stage Rotator Cuff Tendinopathy
Source: Genes (Basel). 2023 Feb 15;14(2):496. doi: 10.3390/genes14020496 (PMC9956879; doi:10.3390/genes14020496)
Supplement: Supplementary file 1 [file genes-14-00496-s001.zip › genes-2184094-supplementary.pdf]

Supplementary Materials

Table S1: Lysis Buffer (500μl)

Reagents Required	Required Volume (μl)
HEPES (0.5M)	20
10% SDS	50
Protease Inhibitor (ThermoFisher 1862209)	5
NaBut (1M)	5
dH_2O	420

Table S2: Decrosslinking Solution

Reagents Required	Required Volume (μl)
Chromatin	5 – 10
NaCl (5M)	4
Proteinase K (20μg/ml)	8
RNase (10mg/ml; ThermoFisher EN0531)	4
dH_2O	85

Table S3: Elution Buffer

Stock Solution	Final Concentration	Required Volume
10% SDS	0.5%	50μl
5M NaCl	300mM	60μl
0.5M EDTA	5mM	10μl
1M Tris-HCl (pH 8.0)	10mM	10μl
dH_2O		870μl

Table S4: Preparation of Invitrogen Dynabeads for Immunoprecipitation

20µl per chromatin sample of beads (Invitrogen Dynabeads Protein A for Immunoprecipitation; 10001D) was taken and placed into 1.5ml Eppendorfs which was topped up to 500µl with dilution buffer including SDS and 0.1% BSA. Beads were then placed into a magnetic rack for 1 minute, or until solution was clear, and the supernatant was then removed. This process was repeated three times, resuspending the beads in 500µl of dilution buffer including SDS and 0.1% BSA every time. Beads were then resuspended in 400µl of dilution buffer including SDS and 0.1% BSA and the antibodies H3K27me3 (Cell Signalling; 9733), H3K4me3 (Cell Signalling; 97515) and H4K20me3 (Cell Signalling; 5737) were then added at a volume of 5µl equating to 1.5µg per chromatin sample and then rotated for 1 hour at RT.

Table S5: Dilution Buffer (with SDS)

Stock Solution	Final Concentration	Required Volume
10% SDS	0.01%	100µl
10% Triton x-100	1%	1ml
0.5M EDTA	1.2mM	24µl
1M Tris-HCl (pH 8.0)	16.7mM	167µl
5M NaCl	167mM	334µl
BSA	0.1%	133µl
dH_2O		8.3ml

Table S6: Dilution Buffer (without SDS)

Stock Solution	Final Concentration	Required Volume
10% Triton x-100	1%	1ml
0.5M EDTA	1.2mM	24µl
1M Tris-HCl (pH 8.0)	16.7mM	167µl
5M NaCl	167mM	334µl
dH_2O		8.3ml

Table S7: Wash Buffer (1)

Stock Solution	Final Concentration	Required Volume
0.5M EDTA	2mM	40µl
1M Tris-HCl (pH 8.0)	20mM	200µl
10% Triton x-100	1%	1ml
10% SDS	0.1%	100µl
5M NaCl	150mM	300µl
dH_2O		8.36ml

Table S8: Wash Buffer (2)

Stock Solution	Final Concentration	Required Volume
0.5M EDTA	2mM	40µl
1M Tris-HCl (pH 8.0)	20mM	200µl
10% Triton x-100	1%	1ml
10% SDS	0.1%	100µl
5M NaCl	500mM	1ml
dH_2O		7.3ml

Table S9: Wash Buffer (3)

Stock Solution	Final Concentration	Required Volume
0.5M EDTA	1mM	20µl
1M Tris-HCl (pH 8.0)	10mM	100µl
dH_2O		9.9ml

Table S10: Primer Combinations and Sequence Information for the Sequencing of ChIP-Seq Samples using Illumina NGS (Semitendinosus Samples)

Sample ID	Index ID (i5 primer)	Index ID (i5 primer sequence)	Index ID (i7 primer)	Index ID (i7 primer sequence)
HA8 Input	504	TCAGAGCC	710	TCCGCGAA
HA8 H3K4me3	501	AGGCTATA	705	ATTCAGAA
HA8 H3K27me3	501	AGGCTATA	709	CGGCTATG
HA9 Input	501	AGGCTATA	711	TCTCGCGC
HA9 H3K4me3	502	GCCTCTAT	705	ATTCAGAA
HA9 H3K27me3	502	GCCTCTAT	709	CGGCTATG
HA10 Input	502	GCCTCTAT	711	TCTCGCGC
HA10 H3K4me3	503	AGGATAGG	705	ATTCAGAA
HA10 H3K27me3	503	AGGATAGG	709	CGGCTATG
HA11 Input	503	AGGATAGG	711	TCTCGCGC
HA11 H3K4me3	504	TCAGAGCC	705	ATTCAGAA
HA11 H3K27me3	504	TCAGAGCC	709	CGGCTATG

Table 1: Dual Primer (NEB; E7600) combinations used for Illumina sequencing of chromatin obtained through ChIP-Seq from hamstring tendon (Semitendinosus) samples collected from patients undergoing routine arthroscopic anterior cruciate ligament reconstruction surgery.

Table S11: Primer Combinations and Sequence Information for the Sequencing of ChIP-Seq Samples using Illumina NGS (Supraspinatus (RCT) Samples))

Sample ID	Index ID (i5 primer)	Index ID (i5 primer sequence)	Index ID (i7 primer)	Index ID (i7 primer sequence)
RCT8 Input	504	TCAGAGCC	711	TCTCGCGC
RCT8 H3K4me3	501	AGGCTATA	706	GAATTCGT
RCT8 H3K27me3	501	AGGCTATA	710	TCCGCGAA
RCT9 Input	501	AGGCTATA	712	AGCGATAG
RCT9 H3K4me3	502	GCCTCTAT	706	GAATTCGT
RCT9 H3K27me3	502	GCCTCTAT	710	TCCGCGAA
RCT10 Input	502	GCCTCTAT	712	AGCGATAG
RCT10 H3K4me3	503	AGGATAGG	706	GAATTCGT
RCT10 H3K27me3	503	AGGATAGG	710	TCCGCGAA
RCT11 Input	504	TCAGAGCC	701	ATTACTCG
RCT11 H3K4me3	501	AGGCTATA	702	TCCGGAGA
RCT11 H3K27me3	502	GCCTCTAT	702	TCCGGAGA
RCT12 Input	504	TCAGAGCC	702	TCCGGAGA
RCT12 H3K4me3	503	AGGATAGG	703	CGCTCATT
RCT12 H3K27me3	502	GCCTCTAT	703	CGCTCATT

Table 2: Dual Primer (NEB; E7600) combinations used for Illumina sequencing of chromatin obtained through ChIP-Seq from diseased supraspinatus samples collected from arthroscopic shoulder surgery to repair rotator cuff tears.

Table S12: Homer Data from Peak Annotation of Significant Peaks Identified for H3K4me3					
**Gene Name**	**Nearest Ensembl**	**Gene Description**	**Gene Type**	**Annotation**	**Distance to TSS**
SHROOM3	ENSG00000138771	shroom family member 3	protein-coding	Intron (1 of 10)	1052
PHF14	ENSG00000106443	PHD finger protein 14	protein-coding	Intergenic	220260
LOC100240735	ENSG00000250654	uncharacterized LOC100240735	ncRNA	Intron (1 of 2)	1143
SIM2	ENSG00000159263	SIM bHLH transcription factor 2	protein-coding	Intron (1 of 9)	1320
DKK2	ENSG00000155011	dickkopf WNT signalling pathway inhibitor 2	protein-coding	Intergenic	-174945
POLRMT	ENSG00000099821	RNA polymerase mitochondrial	protein-coding	TTS	17307
LINC01476	ENSG00000265313	long intergenic non-protein coding RNA 1476	ncRNA	Intron (2 of 2)	26312
JAG2	ENSG00000184916	jagged canonical Notch ligand 2	protein-coding	5' UTR (Exon 1 of 26)	273
CAMK2B	ENSG00000058404	calcium/calmodulin dependent protein kinase II beta	protein-coding	Intron (1 of 18)	351
DACT2	ENSG00000164488	dishevelled binding antagonist of beta catenin 2	protein-coding	Intron (1 of 5)	400
MCF2L	ENSG00000126217	MCF.2 cell line derived transforming sequence like	protein-coding	Intron (4 of 27)	20212
CTAGE1	ENSG00000212710	cutaneous T cell lymphoma-associated antigen 1	protein-coding	Intergenic	69187
CELF2	ENSG00000048740	CUGBP Elav-like family member 2	protein-coding	promoter-TSS	-157

HMX1	ENSG00000215612	H6 family homeobox 1	protein-coding	promoter-TSS	82
EMX2OS	ENSG00000229847	EMX2 opposite strand/antisense RNA	ncRNA	intron (Intron 1 of 1)	897
MBP	ENSG00000197971	Myelin basic protein	protein-coding	intron (Intron 1 of 3)	2083
GALR3	ENSG00000128310	Galanin receptor 3	protein-coding	Exon (Exon 2 of 2)	1581
SMOC2	ENSG00000112562	SPARC related modular calcium binding 2	protein-coding	intron (Intron 1 of 12)	357
TAFA5	ENSG00000219438	TAFA chemokine like family member 5	protein-coding	promoter-TSS	-228
AJAP1	ENSG00000196581	Adherens junctions associated protein 1	protein-coding	5' UTR (Exon 1 of 6)	695
MSX2	ENSG00000120149	msh homeobox 2	protein-coding	Intergenic	-3339
AGAP3	ENSG00000133612	ArfGAP with GTPase domain, ankyrin repeat and PH domain 3	protein-coding	exon (1 of 9)	462
CRMP1	ENSG00000072832	Collapsin response mediator protein 1	protein-coding	exon (1 of 14)	219
PCSK6	ENSG00000140479	Proprotein convertase subtilisin/kexin type 6	protein-coding	exon (1 of 20)	292
CDH4	ENSG00000179242	Cadherin 4	protein-coding	intron (1 of 15)	1104

S12: Simplified Table received from running Homer on the 25 significant peaks identified for H3K4me3 using the DESeq2 data. Here, 25 genes were identified as being nearby to a significant peak, with 22 of these being protein-coding genes, and 3 being ncRNAs.

Table S13: Homer Data from Peak Annotation of Significant Peaks Identified for H3K27me3 (DESeq2)

Gene Name	Nearest Ensembl	Gene Description	Gene Type	Annotation	Distance to TSS
EMBP1	ENSG00000231752	embigin pseudogene 1	Pseudo	Intergenic	1311156
NKX3-2	ENSG00000109705	NK3 homeobox 2	protein-coding	TTS	4340
LOC101927151	ENSG00000267575	uncharacterized LOC101927151	ncRNA	Intergenic	-1688017
FAM230C	ENSG00000279516	family with sequence similarity 230 member C	ncRNA	Intergenic	-1114828
LOC101927050		lysine methyltransferase 2C pseudogene	Pseudo	Intergenic	-199427
NTN1	ENSG00000065320	netrin 1	protein-coding	Exon (2 of 7)	1437
FAM230C	ENSG00000279516	family with sequence similarity 230 member C	ncRNA	Intergenic	-842732
CWH43	ENSG00000109182	cell wall biogenesis 43 C-terminal homolog	protein-coding	Intergenic	169851
CWH43	ENSG00000109182	cell wall biogenesis 43 C-terminal homolog	protein-coding	Intergenic	653828
SPIN4	ENSG00000186767	spindlin family member 4	protein-coding	Intergenic	852862
SIM2	ENSG00000159263	SIM bHLH transcription factor 2	protein-coding	Intergenic	-2423
LOC441666	ENSG00000215146	zinc finger protein 91 pseudogene	Pseudo	Intergenic	455850
ALG10B	ENSG00000175548	ALG10 alpha-1,2-glucosyltransferase B	protein-coding	Intergenic	-1051132
ANKRD30BP2		ankyrin repeat domain 30B pseudogene 2	Pseudo	Intergenic	-1003024
HCN1	ENSG00000164588	hyperpolarization activated cyclic nucleotide gated potassium channel 1	protein-coding	Intergenic	-1988254
FRG1CP	ENSG00000282826	FSHD region gene 1 family member C, pseudogene	Pseudo	Intergenic	104766
LOC441666	ENSG00000215146	zinc finger protein 91 pseudogene	Pseudo	Intergenic	478902
FRG1CP	ENSG00000282826	FSHD region gene 1 family member C, pseudogene	Pseudo	Intergenic	-42356
EMBP1	ENSG00000231752	embigin pseudogene 1	Pseudo	Intergenic	1539728
CWH43	ENSG00000109182	cell wall biogenesis 43 C-terminal homolog	protein-coding	Intergenic	110442
CWH43	ENSG00000109182	cell wall biogenesis 43 C-terminal homolog	protein-coding	Intergenic	167740

HS3ST5	ENSG00000249853	heparan sulfate-glucosamine 3-sulfotransferase 5	protein-coding	Intergenic	-279325
EMB	ENSG00000170571	embigin	protein-coding	Intergenic	782899
CALB1	ENSG00000104327	calbindin 1	protein-coding	Intron (2 of 10)	1462
EMBP1	ENSG00000231752	embigin pseudogene 1	Pseudo	Intergenic	1588509
CWH43	ENSG00000109182	cell wall biogenesis 43 C-terminal homolog	protein-coding	Intergenic	135230
CHEK2P2	ENSG00000259156	checkpoint kinase 2 pseudogene 2	Pseudo	Intergenic	-504131
UBBP4	ENSG00000263563	ubiquitin B pseudogene 4	Pseudo	Intergenic	-229366
CHEK2P2	ENSG00000259156	checkpoint kinase 2 pseudogene 2	Pseudo	Intergenic	-506916
LOC101927151	ENSG00000267575	uncharacterized LOC101927151	ncRNA	Intergenic	-1673181
CWH43	ENSG00000109182	cell wall biogenesis 43 C-terminal homolog	protein-coding	Intergenic	142524
EMBP1	ENSG00000231752	embigin pseudogene 1	pseudo	Intergenic	1666792
EMB	ENSG00000170571	embigin	protein-coding	Intergenic	828879
SRCIN1	ENSG00000277363	SRC kinase signalling inhibitor 1	protein-coding	Exon (7 of 19)	43872
ZNF716	ENSG00000182111	zinc finger protein 716	protein-coding	Intergenic	2304926
EMB	ENSG00000170571	embigin	protein-coding	Intergenic	1999657
LOC101927151	ENSG00000267575	uncharacterized LOC101927151	ncRNA	Intergenic	-1705972
HAVCR1P1	ENSG00000268442	hepatitis A virus cellular receptor 1 pseudogene 1	pseudo	Intergenic	-729516
HAVCR1P1	ENSG00000268442	hepatitis A virus cellular receptor 1 pseudogene 1	pseudo	Intergenic	-497416
HCN1	ENSG00000164588	hyperpolarization activated cyclic nucleotide gated potassium channel 1	protein-coding	Intergenic	-1939864
MIR4265	ENSG00000264934	microRNA 4265	ncRNA	Intron (1 of 9)	-58001
HAVCR1P1	ENSG00000268442	hepatitis A virus cellular receptor 1 pseudogene 1	pseudo	Intergenic	-1797201
HCN1	ENSG00000164588	hyperpolarization activated cyclic nucleotide gated potassium channel 1	protein-coding	Intergenic	-1949044
LOC101927050		lysine methyltransferase 2C pseudogene	pseudo	Intergenic	-752912
EPHA3	ENSG00000044524	EPH receptor A3	protein-coding	Intergenic	1382666

ZXDA	ENSG00000198205	zinc finger X-linked duplicated A	protein-coding	Intergenic	-630818
EMB	ENSG00000170571	embigin	protein-coding	Intergenic	807775
LOC101927050		lysine methyltransferase 2C pseudogene	pseudo	Intergenic	-751874
LOC441666	ENSG00000215146	zinc finger protein 91 pseudogene	pseudo	Intergenic	503199
LMTK3	ENSG00000142235	lemur tyrosine kinase 3	protein-coding	Exon (12 of 16)	13494
LOC101927050		lysine methyltransferase 2C pseudogene	pseudo	Intergenic	-750203
LINC02167	ENSG00000261122	long intergenic non-protein coding RNA 2167	ncRNA	Intergenic	2520060
CCNYL3		cyclin Y like 3 (pseudogene)	pseudo	Intergenic	104256
EFNA5	ENSG00000184349	ephrin A5	protein-coding	Intron (1 of 4)	494
GDF7	ENSG00000143869	growth differentiation factor 7	protein-coding	Exon (2 of 2)	3607
EMX2OS	ENSG00000229847	EMX2 opposite strand/antisense RNA	ncRNA	non-coding (Exon 1 of 4)	188
LOC441666	ENSG00000215146	zinc finger protein 91 pseudogene	pseudo	Intergenic	490083
SPIN4	ENSG00000186767	spindlin family member 4	protein-coding	Intergenic	879106
LOC441666	ENSG00000215146	zinc finger protein 91 pseudogene	pseudo	Intergenic	497274
EMX2	ENSG00000170370	empty spiracles homeobox 2	protein-coding	Intron (2 of 3)	-7873
LOC101927050		lysine methyltransferase 2C pseudogene	pseudo	Intergenic	-761276
EMB	ENSG00000170571	embigin	protein-coding	Intergenic	1703811
SPIN4	ENSG00000186767	spindlin family member 4	protein-coding	Intergenic	885606
EMB	ENSG00000170571	embigin	protein-coding	Intergenic	825628
EMB	ENSG00000170571	embigin	protein-coding	Intergenic	1935371
HAVCR1P1	ENSG00000268442	hepatitis A virus cellular receptor 1 pseudogene 1	pseudo	Intergenic	-1355330
LOC101927050		lysine methyltransferase 2C pseudogene	pseudo	Intergenic	-190662
DEFB115	ENSG00000215547	defensin beta 115	protein-coding	Intergenic	-99861
	ENSG00000164588	hyperpolarization activated cyclic nucleotide	protein-coding	Intergenic	-2039062

HCN1		gated potassium channel 1			
ZNF733P	ENSG00000185037	zinc finger protein 733, pseudogene	pseudo	Intergenic	794670
DCUN1D4	ENSG00000109184	defective in cullin neddylation 1 domain containing 4	protein-coding	Intergenic	-735582
LINC01262	ENSG00000250739	long intergenic non-protein coding RNA 1262	ncRNA	Intergenic	-424404
SPATA31A5	ENSG00000276581	SPATA31 subfamily A member 5	protein-coding	Intergenic	-235081
LOC101927151	ENSG00000267575	uncharacterized LOC101927151	ncRNA	Intergenic	-868899
LOC101927151	ENSG00000267575	uncharacterized LOC101927151	ncRNA	Intergenic	-656725
EMBP1	ENSG00000231752	embigin pseudogene 1	pseudo	Intergenic	1484062
LOC101927050		lysine methyltransferase 2C pseudogene	pseudo	Intergenic	-758000
UBBP4	ENSG00000263563	ubiquitin B pseudogene 4	pseudo	Intergenic	-229901
GDF6	ENSG00000156466	growth differentiation factor 6	protein-coding	Intron (1 of 1)	2288
UBBP4	ENSG00000263563	ubiquitin B pseudogene 4	pseudo	Intergenic	-234175
CHEK2P2	ENSG00000259156	checkpoint kinase 2 pseudogene 2	pseudo	Intergenic	-501781
EMB	ENSG00000170571	embigin	protein-coding	Intergenic	2282693
HAVCR1P1	ENSG00000268442	hepatitis A virus cellular receptor 1 pseudogene 1	pseudo	Intergenic	-1063362
BTN3A2	ENSG00000186470	butyrophilin subfamily 3 member A2	protein-coding	Intergenic	-33738
LOC101927151	ENSG00000267575	uncharacterized LOC101927151	ncRNA	Intergenic	-1007435
EMB	ENSG00000170571	embigin	protein-coding	Intergenic	1309158
EMBP1	ENSG00000231752	embigin pseudogene 1	pseudo	Intergenic	2190397
CWH43	ENSG00000109182	cell wall biogenesis 43 C-terminal homolog	protein-coding	Intergenic	149393
LOC101927050		lysine methyltransferase 2C pseudogene	pseudo	Intergenic	-744660
EMBP1	ENSG00000231752	embigin pseudogene 1	pseudo	Intergenic	2579884
CWH43	ENSG00000109182	cell wall biogenesis 43 C-terminal homolog	protein-coding	Intergenic	153082
CWH43	ENSG00000109182	cell wall biogenesis 43 C-terminal homolog	protein-coding	Intergenic	663896
LINC02167	ENSG00000261122	long intergenic non-protein coding RNA 2167	ncRNA	Intergenic	2531604
ALG10B	ENSG00000175548	ALG10 alpha-1,2-glucosyltransferase B	protein-coding	Intergenic	-1079658
DEFB115	ENSG00000215547	defensin beta 115	protein-coding	Intergenic	-33433

POTEA	ENSG00000188877	POTE ankyrin domain family member A	protein-coding	Intergenic	-53734
SPIN4	ENSG00000186767	spindlin family member 4	protein-coding	Intergenic	851906
EMX2OS	ENSG00000229847	EMX2 opposite strand/antisense RNA	ncRNA	Intergenic	-9334
ALG10B	ENSG00000175548	ALG10 alpha-1,2-glucosyltransferase B	protein-coding	Intergenic	-1077016
LOC101927050		lysine methyltransferase 2C pseudogene	pseudo	Intergenic	-746641
SIM2	ENSG00000159263	SIM bHLH transcription factor 2	protein-coding	Intron (1 of 9)	8928
CWH43	ENSG00000109182	cell wall biogenesis 43 C-terminal homolog	protein-coding	Intergenic	723988
LOC441666	ENSG00000215146	zinc finger protein 91 pseudogene	pseudo	Intergenic	493103
EMB	ENSG00000170571	embigin	protein-coding	Intergenic	823535
INHBB	ENSG00000163083	inhibin subunit beta B	protein-coding	Intergenic	-1694
LINC02167	ENSG00000261122	long intergenic non-protein coding RNA 2167	ncRNA	Intergenic	2529990
LOC101927050		lysine methyltransferase 2C pseudogene	pseudo	Intergenic	-759337
LOC101927050		lysine methyltransferase 2C pseudogene	pseudo	Intergenic	-763231
ZNF716	ENSG00000182111	zinc finger protein 716	protein-coding	Intergenic	1081340
LOC101927151	ENSG00000267575	uncharacterized LOC101927151	ncRNA	Intergenic	-1326024
RIPK4	ENSG00000183421	receptor interacting serine/threonine kinase 4	protein-coding	promoter-TSS	-143
EMB	ENSG00000170571	embigin	protein-coding	Intergenic	2265223
EMX2OS	ENSG00000229847	EMX2 opposite strand/antisense RNA	ncRNA	Intergenic	-6972
HCN1	ENSG00000164588	hyperpolarization activated cyclic nucleotide gated potassium channel 1	protein-coding	Intergenic	-2285612
ROCK1	ENSG00000067900	Rho associated coiled-coil containing protein kinase 1	protein-coding	Intergenic	172462
HAVCR1P1	ENSG00000268442	hepatitis A virus cellular receptor 1 pseudogene 1	pseudo	Intergenic	-1185195
MBP	ENSG00000197971	myelin basic protein	protein-coding	Intron (1 of 3)	1059
SPIN4	ENSG00000186767	spindlin family member 4	protein-coding	Intergenic	887614
EMB	ENSG00000170571	embigin	protein-coding	Intergenic	1384575
HAVCR1P1	ENSG00000268442	hepatitis A virus cellular receptor 1 pseudogene 1	pseudo	Intergenic	-1624689

LOC101927151	ENSG00000267575	uncharacterized LOC101927151	ncRNA	Intergenic	-1604413
EMB	ENSG00000170571	embigin	protein-coding	Intergenic	824782
LOC101927151	ENSG00000267575	uncharacterized LOC101927151	ncRNA	Intergenic	-1710614
EMB	ENSG00000170571	embigin	protein-coding	Intergenic	827950
ZNF716	ENSG00000182111	zinc finger protein 716	protein-coding	Intergenic	2452972
CHEK2P2	ENSG00000259156	checkpoint kinase 2 pseudogene 2	pseudo	Intergenic	-498916
LOC101927050		lysine methyltransferase 2C pseudogene	pseudo	Intergenic	-748126
EMBP1	ENSG00000231752	embigin pseudogene 1	pseudo	Intergenic	1928516
CWH43	ENSG00000109182	cell wall biogenesis 43 C-terminal homolog	protein-coding	Intergenic	145178
LOC101927050		lysine methyltransferase 2C pseudogene	pseudo	Intergenic	-745868
HAVCR1P1	ENSG00000268442	hepatitis A virus cellular receptor 1 pseudogene 1	pseudo	Intergenic	-917305
HCN1	ENSG00000164588	hyperpolarization activated cyclic nucleotide gated potassium channel 1	protein-coding	Intergenic	-1715856
EMB	ENSG00000170571	embigin	protein-coding	Intergenic	1121195
CWH43	ENSG00000109182	cell wall biogenesis 43 C-terminal homolog	protein-coding	Intergenic	150356
EMBP1	ENSG00000231752	embigin pseudogene 1	pseudo	Intergenic	2419884
DEFB115	ENSG00000215547	defensin beta 115	protein-coding	Intergenic	-91806
SPIN4	ENSG00000186767	spindlin family member 4	protein-coding	Intergenic	851040
OR11H1	ENSG00000130538	olfactory receptor family 11 subfamily H member 1	protein-coding	Intergenic	-1172563
LOC441666	ENSG00000215146	zinc finger protein 91 pseudogene	pseudo	Intergenic	506098
LOC441666	ENSG00000215146	zinc finger protein 91 pseudogene	pseudo	Intergenic	454427
EMB	ENSG00000170571	embigin	protein-coding	Intergenic	818667
LOC441666	ENSG00000215146	zinc finger protein 91 pseudogene	pseudo	Intergenic	500773
ZNF733P	ENSG00000185037	zinc finger protein 733, pseudogene	pseudo	Intergenic	2532518
EMB	ENSG00000170571	embigin	protein-coding	Intergenic	822718
LOC101927050		lysine methyltransferase 2C pseudogene	pseudo	Intergenic	-754529

S13: Simplified table received from running Homer on the 145 significant peaks identified for H3K27me3 using the DESeq2. Here, 45 genes were identified as being nearby to a significant peak, with 29 of these being protein-coding genes, 10 being pseudogenes and 6 being ncRNAs. Additionally, some genes,

such as EMB (embigin) and CWH43 (cell wall biogenesis 43 C-terminal homolog) had multiple peak locations (not shown in simplified data set) identified in intergenic regions of this gene.
